# Supplementary material for: Phosphorylation-mediated interactions with TOPBP1 couple 53BP1 and 9-1-1 to control the G1 DNA damage checkpoint
Source: eLife. 2019 May 28;8:e44353. doi: 10.7554/eLife.44353 (PMC6561707; doi:10.7554/eLife.44353)
Supplement: Supplementary file 1. [file elife-44353-supp1.docx]

**Table S1.  Crystallographic Data Collection and Refinement Statistics.**

**llography**

|  | TOPBP1-BRCT0,1,2 53BP1-pT670 | TOPBP1-BRCT4,5 53BP1-pS366 |
| --- | --- | --- |
| Wavelength | 0.9795 | 0.9762 |
| Resolution range | 69.55  - 2.81 (2.91  - 2.81) | 67.4  - 3.53 (3.656  - 3.53) |
| Space group | P 32 2 1 | P 65 2 2 |
| Unit cell | 84.44 84.44 225.19 90 90 120 | 134.81 134.81 303.02 90 90 120 |
| Total reflections | 115011 (11736) | 1469254 (95465) |
| Unique reflections | 23386 (2287) | 20816 (2034) |
| Multiplicity | 4.9 (5.1) | 70.6 (46.9) |
| Completeness (%) | 100 (100) | 100 (100) |
| Mean I/sigma(I) | 17.91 (2.79) | 21.03 (1.88) |
| Wilson B-factor | 89.66 | 133.55 |
| R-merge | 0.04899 (0.6146) | 0.2346 (2.572) |
| R-meas | 0.0552 (0.6847) | 0.2363 (2.6) |
| CC1/2 | 0.998 (0.883) | 1 (0.637) |
| CC* | 0.999 (0.969) | 1 (0.882) |
| Reflections used in refinement | 23383 (2287) | 20813 (2034) |
| Reflections used for R-free | 1132 (118) | 1047 (96) |
| R-work | 0.2229 (0.3935) | 0.2230 (0.3406) |
| R-free | 0.2595 (0.4497) | 0.2362 (0.3659) |
| CC(work) | 0.947 (0.829) | 0.964 (0.655) |
| CC(free) | 0.896 (0.794) | 0.981 (0.684) |
| Number of non-hydrogen atoms | 4605 | 6078 |
| Protein residues | 565 | 790 |
| RMS(bonds) | 0.004 | 0.023 |
| RMS(angles) | 0.67 | 2.44 |
| Ramachandran favored (%) | 96 | 97.93 |
| Ramachandran allowed (%) | 3.6 | 0.78 |
| Ramachandran outliers (%) | 0.53 | 1.30 |
| Rotamer outliers (%) | 1.1 | 0.00 |
| Clashscore | 2.60 | 12.17 |
| Average B-factor | 98.87 | 137.10 |

Statistics for the highest-resolution shell are shown in parentheses.
